# Validation of a breast cancer risk prediction model based on the key risk factors: family history, mammographic density and polygenic risk

**DOI:** 10.1007/s10549-022-06834-7

**Published:** 2023-02-07

**Authors:** Richard Allman, Yi Mu, Gillian S. Dite, Erika Spaeth, John L. Hopper, Bernard A. Rosner

**Affiliations:** 1 Genetic Technologies Limited, 60–66 Hanover St, Fitzroy, VIC 3065 Australia; 2 Channing Division of Network Medicine, Brigham and Women’s Hospital and Harvard Medical School, Boston, MA USA; 3 Phenogen Sciences Inc, Charlotte, NC USA; 4 Centre for Epidemiology and Biostatistics, Melbourne School of Population and Global Health, The University of Melbourne, Parkville, VIC Australia

**Keywords:** Risk prediction model, Polygenic risk, Mammographic density

## Abstract

**Purpose:**

We compared a simple breast cancer risk prediction model, BRISK (which includes mammographic density, polygenic risk and clinical factors), against a similar model with more risk factors (simplified Rosner) and against two commonly used clinical models (Gail and IBIS).

**Methods:**

Using nested case–control data from the Nurses’ Health Study, we compared the models’ association, discrimination and calibration. Classification performance was compared between Gail and BRISK for 5-year risks and between IBIS and BRISK for remaining lifetime risk.

**Results:**

The odds ratio per standard deviation was 1.43 (95% CI 1.32, 1.55) for BRISK 5-year risk, 1.07 (95% CI 0.99, 1.14) for Gail 5-year risk, 1.72 (95% CI 1.59, 1.87) for simplified Rosner 10-year risk, 1.51 (95% CI 1.41, 1.62) for BRISK remaining lifetime risk and 1.26 (95% CI 1.16, 1.36) for IBIS remaining lifetime risk. The area under the receiver operating characteristic curve (AUC) was improved for BRISK over Gail for 5-year risk (*AUC* = 0.636 versus 0.511, *P* < 0.0001) and for BRISK over IBIS for remaining lifetime risk (*AUC* = 0.647 versus 0.571, *P* < 0.0001). BRISK was well calibrated for the estimation of both 5-year risk (expected/observed [*E/O*] = 1.03; 95% CI 0.73, 1.46) and remaining lifetime risk (*E/O* = 1.01; 95% CI 0.86, 1.17). The Gail 5-year risk (*E/O* = 0.85; 95% CI 0.58, 1.24) and IBIS remaining lifetime risk (*E/O* = 0.73; 95% CI 0.60, 0.87) were not well calibrated, with both under-estimating risk. BRISK improves classification of risk compared to Gail 5-year risk (*NRI* = 0.31; standard error [*SE*] = 0.031) and IBIS remaining lifetime risk (*NRI* = 0.287; *SE* = 0.035).

**Conclusion:**

BRISK performs better than two commonly used clinical risk models and no worse compared to a similar model with more risk factors.

**Supplementary Information:**

The online version contains supplementary material available at 10.1007/s10549-022-06834-7.

## Background

For decades, women have been told that they have an increased risk of breast cancer if they have a family history of the disease [1], and more recently, women have been told that they have increased risk of breast cancer if they have mammographically dense breasts [2]. Genetic risk derived from common variants throughout the genome can also be used to predict risk of breast cancer [3] but this information is not readily available to women. This fragmented approach to risk prediction can result in women being given inaccurate risk information and can mean that women get conflicting and confusing advice.

Our goal is to accurately predict breast cancer risk for unaffected women by combining information on the key risk factors (including polygenic risk, mammographic density and family history) into a single simple model rather than by considering the risk factors separately [4–6]. This approach is of practical importance for both clinical and population health. In clinical practice, women at very high risk of breast cancer can be identified and given risk reduction options including medication or alternative screening modalities. While clinical implementation of reduced screening is not yet standard of care, women at very low risk of breast cancer can be identified as candidates for less frequent screening, with a concomitant financial benefit to population-based screening programs.

About 15% of women report having at least one first-degree relative with breast cancer [7–10], and a further 20% report having at least one affected second-degree relative [11]. The magnitude of familial risk depends on the closeness of the genetic relationship, the age at diagnosis of the affected relative and the age of the at-risk woman [1]. Familial risk is best captured by algorithms that model major genes and underlying genetic risk using multi-generational pedigree data [12–15]. Cancer family clinics use these tools when managing the small proportion of women who have several affected relatives and where there is the time and commitment to collect and validate extensive family cancer data. This approach is not applicable on a population basis.

Deeper understanding of breast cancer family history based on genetics led to the discovery of the first major susceptibility genes for breast cancer (*BRCA1* and *BRCA2*) over 20 years ago [16]. Since then, additional highly and moderately penetrant risk variants have been identified in other genes [16]. These hereditary high-risk variants are rare. For example, *BRCA1* and *BRCA2* variants are found in only 1 in 400 women [17], but are associated with a full lifetime breast cancer risk of over 50% [18]. While it is important to identify women with these variants, they represent only about 5% of breast cancer cases [19]. The rarity of these mutations means they have limited impact on population health.

There are genetic implications at the population level if we look beyond highly or moderately penetrant variants. Large collaborative studies have identified single-nucleotide polymorphisms (SNPs) that are independently associated with breast cancer risk [20–22]. The breast cancer associations of these SNPs are small, with the odds ratio (OR) per risk allele typically being 1.1 or less [22], and empirically these risks appear to multiply [23]. These SNPs can be used to create a polygenic risk score (PRS) that can predict risk of breast cancer. Mavaddat et al. [3] first identified a 77-SNP PRS that has an OR per standard deviation (SD) of 1.46. More recently, Mavaddat et al. [24] identified a 313-SNP PRS that has an OR per SD of 1.61.

Mammographic density, visualized by the bright regions on a mammogram, can be measured on a continuous scale using a computer-assisted method (e.g. Cumulus) [25] or a fully automated method (e.g. Volpara) [26]. Mammographic density can also be assigned to categories by radiologists after visual inspection (e.g. BI-RADS classification) [27]. Percent mammographic density (adjusted for age and body mass index) is associated with risk of breast cancer [28]. Over 40% of women in the USA have dense breasts, more so at young ages [2]. Unfortunately, most women are unaware of risk factors other than family history and there is limited and often conflicting guidance for healthcare providers to recommend alternative screening modalities based on mammographic density alone.

These three major risk factors for breast cancer, and other risk factors such as body mass index and menopausal status, act largely independently [29]. Therefore, in terms of absolute risk, the combination of risk factors is extremely important for accurately determining risk. Information on these risk factors can be collected with a simple questionnaire or from electronic health records, while DNA analysis involves a simple buccal collection. This simplified approach allows for scalability of risk assessment in the general population without affecting the already-limited clinician-patient interaction.

In this paper we use a nested case–control dataset from the Nurses’ Health Study to assess the performance of a new risk model (BRISK) compared with the simplified Rosner model [30] and the standard clinical models Gail [31] and IBIS (version 7) [13]. Traditionally, the Gail model has been used for determining 5-year risks in the general population and IBIS has been used to predict remaining lifetime risk for women with a strong family history. BRISK was designed to combine data that is simple to collect in clinical practice with measures of mammographic density and polygenic risk that can be provided by other services.

## Materials and methods

### BRISK model

The model was constructed by bringing together evidence for the major risk factors for breast cancer. The input variables include age, number of affected first-degree relatives, age of youngest first-degree relative, number of affected second-degree relatives, percent mammographic density (or BI-RADs category), body mass index, and menopausal status. A description of the model is provided in the supplementary data.

The family history risks were based on those in the Collaborative Group on Hormonal Factors in Breast Cancer analysis [1]. These risks were smoothed and centered to have a population average risk of 1. The 313 SNPs in the PRS were those from Mavaddat et al. [24] and were combined to form a PRS using the approach of Mealiffe et al. [32]. The estimates for body mass index for pre- and post-menopausal women were taken from Hopper et al. [29]. The estimates for percent mammographic density were taken from unpublished analyses of the Australian Breast Cancer Family Registry [33] and the Australian Mammographic Density Twins and Sisters Study [34] and included estimates for percent mammographic density as a continuous measure and an alternative categorical measure to approximate BI-RADS classifications.

### Participants

Our analyses used nested case–control data from the Nurses’ Health Study, which was established in 1976 and included 121,700 female registered nurses aged 30–55 years [35]. Questionnaires were mailed to women biennially to collect information on breast cancer risk factors, including age at menarche, age at first birth, parity, family history of breast cancer, height, weight, menopausal status, age at menopause, and hormone replacement therapy use. The nested case–control dataset comprised 1131 breast cancer cases and 1700 controls for whom questionnaire, mammographic density, and genotyping data were available. A subset of cases (*n* = 881) and controls (*n* = 1327) also had IBIS (version 7) risk predictions available for analysis. A further subset of the cases had estrogen receptor (ER) status available: 562 ER-positive and 106 ER-negative.

### Statistical methods

We used standard univariate methods based on *t*-tests for continuous variables and contingency table methods for categorical variables to compare risk scores and covariates between cases vs. controls with results presented as OR per SD of risk. We used the area under the receiver operating characteristic curve (AUC) to compare the risk score distributions between cases vs. controls [36]. We compared the AUCs for competing risk models using the methods of DeLong [37]. For calibration, we compared the expected number of incident cases over 5 years for controls (median 5-year age-specific BRISK score ✕ number of women in 5-year age groups) to the number of incident cases that would be observed based on 5-year population incidence rates. Calibration was performed using controls because they are representative of the general population. Similar methods were used for estimated remaining lifetime risk. The median (rather than the mean) was used because the age-specific distribution of BRISK 5-year and remaining lifetime risk scores were strongly right-skewed. We used reclassification tables to compare pairs of risk models in terms of their ability to assign women to categories of risk based upon clinical thresholds used to guide chemoprevention or increased screening. National Comprehensive Cancer Network guidelines for breast cancer risk reduction recommend that 5-year risks be assessed using the Gail model, with patients exceeding a threshold of 1.67% be offered risk-reducing medication. The United States Preventive Services Task Force and the American Society of Clinical Oncology use 5-year risk threshold of 3%, based on evidence that women over this threshold are most likely to benefit from endocrine prevention therapy. Therefore, the reclassification for 5-year risk estimation compared Gail versus the BRISK model using both thresholds. Clinical guidelines for breast cancer screening recommend that lifetime risk estimates be assessed using models that are largely based upon family history, with women exceeding a risk threshold of 20% in the USA (or 25% in other countries) to be offered increased screening for breast cancer, therefore the reclassification for lifetime risk estimation compared IBIS versus the BRISK model. We categorized the estimated 5-year risk using categories (< 1%, ≥ 1 to < 1.67%, ≥ 1.67% to < 3%, and ≥ 3%), and used the net reclassification index (NRI) from Pencina and Steyerberg [38] to cross-classify the risk score distribution of alternative risk models, separately for cases and controls [39]. A similar approach was used to cross-classify estimated remaining lifetime risk using the categories (< 6%, ≥ 6% to < 12%, ≥ 12% to < 20%, ≥ 20% to < 25%, and ≥ 25%). NRI analyses for cases were performed for overall breast cancer as well as by ER and PR subtype (ER positive and PR positive; ER negative and PR negative) and stage (stage 1; stage ≥ 2).

## Results

### Participants

The risk factors for cases and controls are summarized in Table 1 and are in broad agreement with previously published data [40, 41].

**Table 1:** Risk factors by case–control status

Variable	Case	Control
	(*N* = 1131)	(*N* = 1700)
Age (years)		
Mean (SD)	58.4 (7.4)	59.3 (7.5)
Median (IQR)	58.0 (53.0, 64.0)	60.0 (54.0, 65.0)
Percent density		
Mean (SD)	32.0 (19.6)	24.7 (18.1)
Median (IQR)	29.2 (15.9, 45.5)	21.0 (10.9, 35.1)
Body mass index (kg/m^2^)		
Mean (SD)	25.9 (4.9)	25.9 (4.7)
Median (IQR)	25.0 (22.3, 28.3)	24.9 (22.5, 28.3)
Race, *N* (%)		
White, non-Hispanic	1125 (99)	1694 (100)
Black, non-Hispanic	1 (0.1)	0 (0)
Hispanic	6 (0.5)	6 (0)
Other	0 (0)	0 (0)
First-degree family history, *N* (%)		
0	932 (82)	1454 (86)
1	178 (16)	233 (14)
≥ 2	22 (2)	13 (0)
Second-degree family history, *N* (%)		
0	1047 (92)	1598 (94)
1	83 (7)	100 (6)
2 or more	2 (0)	2 (0)
Density category, *N* (%)		
< 25%	474 (42)	1018 (60)
≥ 25%	658 (58)	682 (40)
Menopausal status, *N* (%)		
Premenopausal	205 (18)	261 (15)
Postmenopausal	927 (82)	1439 (85)
Age at menarche (years)		
Mean (SD)	12.5 (1.6)	12.6 (1.4)
Median (IQR)	12 (12, 13)	13 (12, 13)
Age at menarche, *N* (%)		
< 12 years	276 (24)	369 (22)
≥ 12 to < 15 years	771 (68)	1186 (70)
≥ 15 years	78 (7)	136 (8)
Parity		
0	83 (7)	99 (6)
1	78 (7)	93 (5)
≥ 2	955 (84)	1499 (88)
Age at 1st live birth (years)^a^		
Mean (SD)	25.3 (3.4)	25.1(3.1)
Median (IQR)	25 (23, 27)	24 (23, 27)
Age at 1st live birth, *N* (%)^a^		
< 20 (years)	5 (0)	7 (0)
≥ 20 to < 25(years)	504 (49)	818 (51)
≥ 25 to < 30 (years)	418 (40)	614 (39)
≥ 30 (years)	106 (10)	153 (10)
Age at menopause (years)^b^		
Mean (SD)	49.7 (4.1)	49.5 (4.3)
Median (IQR)	50 (48, 52)	50 (48, 52)
Age at menopause, *N* (%)^b^		
< 45 (years)	75 (9)	135 (10)
≥ 45 to < 50 (years)	215 (24)	342 (25)
≥ 50 to < 55 (years)	532 (60)	831 (60)
≥ 55 (years)	58 (7)	77 (6)
Hormone replacement therapy use, *N* (%)^b^		
Never	275 (31)	545 (39)
Current user	449 (51)	530 (38)
< 5 years	180 (40)	220 (42)
≥ 5 years	269 (60)	310 (58)
Past user	165 (19)	310 (22)
< 5 years	119 (72)	230 (74)
≥ 5 years	46 (28)	80 (26)
Hormone replacement therapy length of use, *N* (%)^b^		
< 5 years	300 (27)	451 (27)
≥ 5 years	316 (28)	392 (23)
Prior breast biopsy, *N* (%)		
No	836 (74)	1333 (78)
Yes, non-proliferative	14 (5)	12 (3)
Yes, proliferative without atypia	32 (11)	29 (8)
Yes, atypical hyperplasia	11 (4)	1 (0)
Yes, lobular carcinoma in situ or unknown	1 (0)	0 (0)
Yes, missing	238 (80)	325 (89)
Stage at diagnosis, *N* (%)		
Stage I	600 (53)	NA
Stage II	232 (20)	NA
Stage III	69 (6)	NA
Stage IV	1 (0)	NA
Missing	230 (20)	NA
Tumor subtype, *N* (%)		
ER + , PR +	708 (63)	NA
ER − , PR − , HER2 +	41 (4)	NA
ER − , PR − , HER2 −	99 (9)	NA
Missing	284 (25)	NA
Tumor molecular subtype, *N* (%)		
Luminal A	175 (15)	NA
Luminal B	205 (18)	NA
HER2-enriched	41 (4)	NA
Basal-like	33 (3)	NA
Unspecified	11 (1)	NA
Missing	667 (59)	NA
BRISK 5-year risk (Cumulus)		
Mean (SD)	0.054 (0.067)	0.033 (0.048)
Median (IQR)	0.031 (0.015, 0.064)	0.018 (0.009, 0.038)
BRISK 5-year risk (density category)		
Mean (SD)	0.051 (0.064)	0.031 (0.046)
Median (IQR)	0.030 (0.014, 0.062)	0.017 (0.009, 0.036)
BRISK remaining lifetime risk (Cumulus)		
Mean (SD)	0.230 (0.208)	0.144 (0.154)
Median (IQR)	0.162 (0.077, 0.322)	0.091 (0.043, 0.185)
BRISK remaining lifetime risk (density category)		
Mean (SD)	0.218 (0.200)	0.137 (0.149)
Median (IQR)	0.153 (0.073, 0.294)	0.087 (0.041, 0.175)
Gail 5-year risk		
Mean (SD)	0.018 (0.009)	0.017 (0.008)
Median (IQR)	0.016 (0.012, 0.020)	0.015 (0.012, 0.020)
Gail full-lifetime risk		
Mean (SD)	0.090 (0.041)	0.082 (0.033)
Median (IQR)	0.080 (0.065, 0.104)	0.076 (0.063, 0.095)
IBIS (v7 with Cumulus) 5-year risk^c^		
Mean (SD)	0.017 (0.008)	0.016 (0.007)
Median (IQR)	0.015 (0.011, 0.019)	0.014 (0.011, 0.018)
IBIS (v7 with Cumulus) remaining lifetime risk^c^		
Mean (SD)	0.101 (0.042)	0.092 (0.038)
Median (IQR)	0.094 (0.074, 0.118)	0.087 (0.066, 0.108)
Simplified Rosner 10-year risk^d^		
Mean (SD)	2.20 (0.56)	1.90 (0.54)
Median (IQR)	2.16 (1.80, 2.57)	1.85 (1.52, 2.22)

*IQR* inter-quartile range, *SD* standard deviation

^a^Among women who have parity ≥ 1

^b^Post-menopause

^c^For IBIS, *N* = 881 for cases; *N* = 1327 for controls

^d^For simplified Rosner, *N* = 1088 for cases; *N* = 1640 for controls

### Association and discrimination

The OR per SD and AUC for each of the models are presented in Table 2. Discrimination was improved for BRISK 5-year risk over Gail 5-year risk (*χ*^2^ = 88.29, degrees of freedom [*d.f.*] = 1, *P* < 0.0001) and for BRISK remaining lifetime risk over IBIS remaining lifetime risk (*χ*^2^ = 22.91, *d.f.* = 1, *P* < 0.0001). There was no difference in AUC between the simplified Rosner 10-year risk and either BRISK 5-year risk (*χ*^2^ = 3.69, *d.f.* = 1, *P* = 0.06) or BRISK remaining lifetime risk (*χ*^2^ = 1.18, *d.f.* = 1, *P* = 0.3). Similar results were obtained for the alternate specification of BRISK that uses approximated BI-RADS categories for the mammographic density measure (see Supplementary Table 1).

**Table 2:** Performance of 5-year and lifetime risk calculation by the BRISK model and compared to the commonly used Gail and IBIS models and another recently developed model (simplified Rosner score) that includes more risk factors than the BRISK risk model

Model	OR per SD^a^	95% CI	P	AUC	95% CI
BRISK 5-year risk	1.43	1.32, 1.55	< 0.0001	0.636	0.615, 0.657
BRISK remaining lifetime risk	1.51	1.41, 1.62	< 0.0001	0.647	0.627, 0.668
Gail 5-year risk	1.07	0.99, 1.14	0.079	0.511	0.489, 0.533
IBIS v7 remaining lifetime risk^b^	1.26	1.16, 1.36	< 0.0001	0.571	0.546, 0.595
Simplified Rosner 10-year risk^c^	1.72	1.59, 1.87	< 0.0001	0.657	0.636, 0.678

*AUC* area under receiver operating characteristic curve, *CI* confidence interval, *OR* odds ratio, *SD* standard deviation

^a^Per standard deviation in controls

^b^For IBIS, *N* = 881 for cases; *N* = 1327 for controls

^c^For simplified Rosner, *N* = 1088 for cases; *N* = 1640 for controls

### Model calibration

**Fig. 1:**
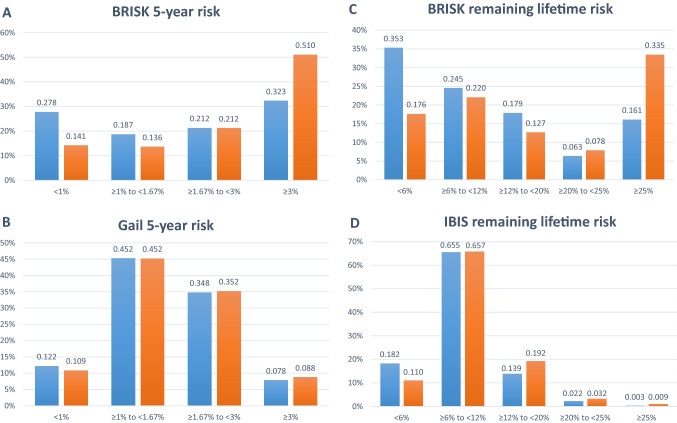
Distribution of risk scores at clinically relevant thresholds for controls (blue) and cases (orange). **A** BRISK 5-year risk, **B** Gail 5-year risk, **C** BRISK remaining lifetime risk, **D** IBIS remaining lifetime risk

BRISK was very well calibrated for the estimation of both 5-year risk (*E/O* = 1.03; 95% CI 0.73, 1.46) and remaining lifetime risk (*E/O* = 1.01; 95% CI 0.86, 1.17). The Gail 5-year risk (*E/O* = 0.85; 95% CI 0.58, 1.24) and IBIS remaining lifetime risk estimates (*E/O* = 0.73; 95% CI 0.60, 0.87) were not well calibrated in this dataset, with both significantly under-estimating risk (Table 3). The distributions of cases and controls according to the 5-year or lifetime risk estimates provided by the relevant model are presented in Fig. 1, demonstrating separation of controls (orange) to the left and cases (blue) to the right. The data illustrate the assignment of controls to lower risk and the concomitant assignment of cases to higher risk by the BRISK model compared to Gail and IBIS.

**Table 3:** Calibration of 5-year and remaining lifetime risk estimates for cases obtained using the BRISK model versus Gail 5-year risk and IBIS version 7 remaining lifetime risk

Age group (years)	*N*	BRISK 5-year expected	BRISK 5-year observed			Gail 5-year expected	Gail 5-year observed
< 45	24	0.32	0.23			0.20	0.23
45–49	180	2.27	2.06			1.70	2.06
50–54	273	4.13	3.58			3.19	3.58
55–59	342	6.89	5.30			4.80	5.30
60–64	441	9.13	8.76			7.67	8.76
65–69	317	6.51	7.68			6..02	7.68
70–74	97	1.74	2.50			1.90	2.52
75 +	26	0.78	0.63			0.53	0.63
Total	1700	31.78	30.74			26.01	30.75
E/O Ratio		1.03				0.85	
E/O Ratio 95% CI		(0.73, 1.46)				(0.58, 1.24)	

Age group (years)	*N*	BRISK remaining lifetime expected	BRISK remaining lifetime observed	IBIS v7 remaining lifetime expected	IBIS v7 remaining lifetime observed
< 45	24	4.28	3.23	2.78	3.58
45–49	180	23.67	22.90	17.25	23.24
50–54	273	32.54	32.28	22.64	30.32
55–59	342	41.10	35.96	23.79	33.26
60–64	441	37.52	39.54	26.94	38.01
65–69	317	18.78	22.12	14.49	20.30
70–74	97	3.33	4.42	3.62	5.11
75 +	26	0.60	0.51	0.66	0.76
Total	1700	161.83	160.96	112.18	154.59
E/O Ratio		1.01		0.73	
E/O Ratio 95% CI		(0.86, 1.17)		(0.60, 0.87)	

*CI* confidence interval, *E* expected, *O* observed

### Classification performance

5-year risk

We used reclassification tables to assess the assignment of women to the following 5-year risk categories: < 1%, > 1 to < 1.67%, > 1.67 to < 3% and > 3%, which cover the two most widely used thresholds for offering chemopreventive medication. For 5-year risk, BRISK improved classification performance for cases and controls over Gail 5-year risk with an NRI of 0.31 (standard error [*SE*] = 0.031). The reclassification improvement for cases was 0.415 (*SE* = 0.023) and − 0.103 (*SE* = 0.021) for controls (Table 4). The BRISK model assigned 72.2% of cases above the 1.67% National Comprehensive Cancer Network threshold for risk-reducing medication compared to 43.9% by Gail. At the United States Preventive Services Task Force threshold of 3%, the BRISK model assigned 51% of cases above the threshold, with only 8.75% being identified at this high-risk category by Gail (Supplementary Table 2).

The classification performance in cases was consistent for both ER positive cases (reclassification improvement = 0.44, SE 0.03) and ER negative cases (reclassification improvement = 0.31, SE 0.07) (Tables 5 and 6). The BRISK model assigned 53% of ER positive cases and 39.9% of ER negative cases above the 3% 5-year risk threshold.

Remaining lifetime risk

We also used reclassification tables to assess the assignment of women to the following lifetime risk categories: < 6%, > 6 to < 12%, > 12 to < 20%, > 20 to < 25% and > 25%, which cover the two most widely used thresholds for offering increased screening by MRI. BRISK remaining lifetime risk improved classification performance for cases and controls over IBIS remaining lifetime risk with an overall NRI of 0.287 (*SE* = 0.035). The reclassification improvement for cases was 0.381 (*SE* = 0.026) and − 0.094 (*SE* = 0.022) for controls (Table 7). The BRISK model assigned 41.3% of cases above the 20% National Comprehensive Cancer Network lifetime risk threshold for offering MRI screening compared to just 4.1% by IBIS. At the higher threshold of 25%, the BRISK model assigned 33.5% of cases above the threshold, with only 0.9% being identified at this high-risk category by IBIS (Supplementary Table 3). The improved classification performance in cases was consistent for both ER positive cases (*reclassification improvement* = 0.425; *SE* = 0.032) and ER negative cases (*reclassification improvement* = 0.34; *SE* = 0.079; Tables 8 and 9). The reclassification of lifetime risk is also favorable when analyzed by stage at diagnosis with the reclassification improvement for stage 1 *cases* = 0.397 (*SE* = 0.036) and for later stages (2 +) *reclassification improvement* = 0.423 (*SE* = 0.050; Tables 10 and 11).

**Table 4:** Reclassification for Gail 5-year risk versus BRISK 5-year risk

Gail 5-year risk vs BRISK 5-year risk						
Cases		BRISK 5-year risk				
		< 1%	≥ 1 to < 1.67%	≥ 1.67 to < 3%	≥ 3%	Total
Gail 5-year risk	< 1%	25	28	31	39	123
	≥ 1 to < 1.67%	92	70	115	234	511
	≥ 1.67 to < 3%	37	54	79	228	398
	≥ 3%	6	2	15	76	99
	Total	160	154	240	577	1131

Controls		BRISK 5-year risk				
		< 1%	≥ 1 to < 1.67%	≥ 1.67 to < 3%	≥ 3%	Total
Gail 5-year risk	< 1%	96	34	41	36	207
	≥ 1 to < 1.67%	231	146	163	229	769
	≥ 1.67 to < 3%	131	117	134	209	591
	≥ 3%	14	21	23	75	133
	Total	472	318	361	549	1700

NRI (SE): overall = 0.31 (0.031). Classification improvement for cases = 0.415 (0.023), classification improvement for controls = −0.103 (0.021)

**Table 5:** Reclassification for Gail 5-year risk versus BRISK 5-year risk for ER + and PR + cases

ER + and PR + : Gail 5-year risk vs BRISK 5-year risk						
Cases		BRISK 5-year risk				
		< 1%	≥ 1 to < 1.67%	≥ 1.67 to < 3%	≥ 3%	Total
Gail 5-year risk	< 1%	15	11	15	32	73
	≥ 1 to < 1.67%	50	47	77	162	336
	≥ 1.67 to < 3%	22	33	51	129	235
	≥ 3%	3	1	7	52	63
	Total	90	92	150	375	707

Classification improvement (*SE*) = 0.438 (0.029)

**Table 6:** Reclassification for Gail 5-year risk versus BRISK 5-year risk for ER − and PR − cases

ER − and PR − : Gail 5-year vs BRISK 5-year						
Cases		BRISK 5-year risk				
		< 1%	≥ 1 to < 1.67%	≥ 1.67 to < 3%	≥ 3%	Total
Gail 5-year risk	< 1%	5	8	7	5	25
	≥ 1 to < 1.67%	14	9	12	18	53
	≥ 1.67 to < 3%	4	8	10	27	49
	≥ 3%	1	1	5	6	13
	Total	24	26	34	56	140

Classification improvement (*SE*) = 0.314 (0.070)

**Table 7:** Reclassification for IBIS lifetime risk versus BRISK lifetime risk

Cases		BRISK lifetime risk					
		< 6%	≥ 6 to < 12%	≥ 12 to < 20%	≥ 20 to < 25%	≥ 25%	Total
IBIS v7 lifetime risk	< 6%	38	32	11	3	13	97
	≥ 6 to < 12%	98	122	117	49	193	579
	≥ 12 to < 20%	17	37	30	16	69	169
	≥ 20 to < 25%	2	3	9	1	13	28
	≥ 25%	0	0	1	0	7	8
	Total	155	194	168	69	295	881

Controls		BRISK lifetime risk					
		< 6%	≥ 6 to < 12%	≥ 12 to < 20%	≥ 20 to < 25%	≥ 25%	Total
IBIS v7 lifetime risk	< 6%	132	46	37	8	18	241
	≥ 6 to < 12%	292	235	153	59	130	869
	≥ 12 to < 20%	40	43	40	15	46	184
	≥ 20 to < 25%	4	1	6	2	16	29
	≥ 25%	0	0	1	0	3	4
	Total	468	325	237	84	213	1327

NRI (*SE*): overall = 0.287 (0.035). Classification improvement for cases = 0.381 (0.026), classification improvement for controls = −0.094 (0.022)

**Table 8:** Reclassification for IBIS lifetime risk versus BRISK lifetime risk for ER + and PR + cases

Cases		BRISK lifetime risk					
		< 6%	≥ 6 to < 12%	≥ 12 to < 20%	≥ 20 to < 25%	≥ 25%	Total
IBIS v7 lifetime risk	< 6%	29	23	5	2	8	67
	≥ 6 to < 12%	53	74	72	31	136	366
	≥ 12 to < 20%	11	22	21	9	44	107
	≥ 20 to < 25%	2	3	5	1	6	17
	≥ 25%	0	0	1	0	4	5
	Total	95	122	104	43	198	562

Classification improvement (*SE*) = 0.425 (0.0324)

**Table 9:** Reclassification for IBIS lifetime risk versus BRISK lifetime risk for ER − and PR − cases

Cases		BRISK lifetime risk					
		< 6%	≥ 6 to < 12%	≥ 12 to < 20%	≥ 20 to < 25%	≥ 25%	Total
IBIS v7 lifetime risk	< 6%	5	3	3	0	4	15
	≥ 6 to < 12%	11	15	15	8	12	61
	≥ 12 to < 20%	4	7	4	4	8	27
	≥ 20 to < 25%	0	0	1	0	2	3
	≥ 25%	0	0	0	0	0	0
	Total	20	25	23	12	26	106

Classification improvement (*SE*) = 0.340 (0.079)

**Table 10:** Reclassification for IBIS lifetime risk versus BRISK lifetime risk for Stage 1 cases

Cases		BRISK lifetime risk					
		< 6%	≥ 6 to < 12%	≥ 12 to < 20%	≥ 20 to < 25%	≥ 25%	Total
IBIS v7 lifetime risk	< 6%	25	17	4	1	6	53
	≥ 6 to < 12%	48	65	57	29	109	308
	≥ 12 to < 20%	9	17	17	5	35	83
	≥ 20 to < 25%	2	3	4	1	6	16
	≥ 25%	0	0	1	0	5	6
	Total	84	102	83	36	161	466

Classification improvement (*SE*) = 0.397 (0.036)

**Table 11:** Reclassification for IBIS lifetime risk versus BRISK lifetime risk for Stage 2 + cases

Cases		BRISK lifetime risk					
		< 6%	≥ 6 to < 12%	≥ 12 to < 20%	≥ 20 to < 25%	≥ 25%	Total
IBIS v7 lifetime risk	< 6%	9	11	4	1	5	30
	≥ 6 to < 12%	23	35	37	12	44	151
	≥ 12 to < 20%	3	15	7	7	20	52
	≥ 20 to < 25%	0	0	2	0	3	5
	≥ 25%	0	0	0	0	1	1
	Total	35	61	50	20	73	239

Classification improvement (*SE*) = 0.423 (0.050)

## Discussion

Many models have been developed to estimate a women’s risk of developing breast cancer; these can be summarized as simple clinical models such as the Gail model [31], which was designed for the general population, comprehensive models such as simplified Rosner [30], or complex pedigree-based models designed for use in a familial genetics setting such as IBIS [13]. In this paper, we have assessed the performance of a new risk model, BRISK, which incorporates family history, body mass index, menopausal status, polygenic risk and mammographic density in a format that does not require a complex pedigree assessment or multi-page questionnaire. The DNA required for genotyping can readily be obtained by buccal or saliva sample. This simple design specification is important in a general practice or mammography clinic setting where patient contact time is necessarily short, but where a risk assessment is useful and actionable. This concept is supported by developments in colorectal cancer risk assessment where reducing the questionnaire complexity has also been shown to not adversely affect model performance, but does increase ease of use [42–45].

We used a nested case–control dataset from the Nurses’ Health Study to compare the model’s performance to existing models. Our results are consistent with studies that have investigated the association of mammographic density and PRS with breast cancer risk [46–48], including those that have incorporated a risk prediction model [49, 50]. We used the classic version of IBIS (without SNPs or mammographic density) because this was the only version available in the Nurses’ Health Study. We aim to compare BRISK against IBIS version 8 at the earliest opportunity.

Overall, BRISK has superior discrimination and is better calibrated than both Gail (for 5-year risk) and IBIS (for remaining lifetimes risk), which are in widespread clinical usage (Tables 2, 3, 4). BRISK identifies 51% of cases and 32% of controls who are over the United States Preventive Services Task Force 5-year risk threshold where chemoprevention is recommended. BRISK also identifies 41% of cases and 22% of controls who are over the National Comprehensive Cancer Network 20% remaining lifetime risk threshold where increased screening would be offered.

Reclassification analysis of both 5-year and remaining lifetime risk suggested that the BRISK model improves classification for both ER positive and ER negative disease; however, the model still better predicts ER-positive disease. The SNPs in this model have ER-dependent ORs that, if applied, might further improve the tumor subtype prediction. We aim to further validate BRISK in this capacity because it could have even greater implications on clinician recommendation and patient uptake of risk-reducing medication and could enable future risk reduction options for those with ER-negative disease. Limitations to risk assessment remain despite improvements upon current standards shown within these data; the model is still a prediction. We still categorize a proportion of women within average risk categories (< 1.67%) who still go on to develop breast cancer, suggesting there remain unknown risk factors that can contribute to risk that models currently do not capture. However, the enhancement of current risk stratification is significant, and provides impactful clinical benefit.

When considering population level risk reduction measures, the proportion of the population identified as at-risk is reasonable. If used in clinical practice the model would identify between a third and a fifth of women aged 40–70 years as being at increased risk, either by 5-year risk (≥ 3.0%) or remaining lifetime risk (≥ 20%), respectively, meaning that we would subject approximately one-third of all women to increased surveillance or risk-reducing discussions with a clinician. This is a substantial increase over the current 2–10% identified by Gail or IBIS.

Risk-reducing medication is effective at reducing 38–65% of breast cancer incidence depending on the selective ER modulator or aromatase inhibitor clinical trial [51–55]. Despite having several risk-reducing medications that have been shown to reduce the risk of breast cancer, uptake has been dismal [56]. If risk-reducing medication recommendation could be improved through the use of a risk prediction model such as BRISK, a significant reduction in breast cancer incidence could be achieved.

Implications for screening modifications based on the BRISK model at 20% or 25% actionable thresholds for at-risk women are substantial. Based on this case–control study, we show that BRISK is capable of identifying equivalent proportions of stage 1 and stage 2 + breast cancer cases above an actionable risk threshold (Supplementary Table 3. These data suggest that if the stage 2 + women had been assessed with BRISK prior to diagnosis, they would have been identified as at-risk and provided additional screening based on their actionable risk level; they therefore could have been diagnosed with an earlier stage breast cancer.

Screening and risk-reducing options exist for breast cancer; however, implementation of the already accessible tools remains a challenge. Stratification of the general population is the first step towards enabling a more structured conversation on risk reductions for an at-risk woman, whether it be centered on lifestyle habits, surveillance or medication. Furthermore, inconsistent medical body recommendations on screening mammography and mammographic density notification can make it challenging for clinicians to make decisions, especially at a primary care level when they are required to know screening guidance for a wide variety of diseases. Enabling a basic conversation about breast cancer risk with the help of a simple risk model can support clinician use of risk assessment without the burden of specialized knowledge in the area.

In summary, we have developed an easy to implement risk model that enables clinicians to have increased visibility of at-risk patients compared with current clinical models, independent of the estrogen receptor status of future tumors or potential aggressive nature of the disease. This risk model was constructed with real-world implementation concerns in mind. It incorporates only the most impactful epidemiological factors. Focusing on a few risk factors that are simple to collect will save time and remove potential ambiguity due to incomplete questionnaires. Ultimately this improves risk stratification beyond current model performance without impeding on physician time management.

## Supplementary Information

Below is the link to the electronic supplementary material.

Supplementary file1 (DOCX 21 KB)

## Data Availability

The data generated in this study are not publicly available due to participant confidentiality and privacy concerns but are available upon request. Data access must be approved by the institutional review boards of the Brigham and Women’s Hospital and Harvard T.H. Chan School of Public Health. Further information including the procedures to obtain and access data from the Nurses’ Health Studies is described at: https://www.nurseshealthstudy.org/researchers.
