# Supplementary material for: Validation of a breast cancer risk prediction model based on the key risk factors: family history, mammographic density and polygenic risk
Source: Breast Cancer Res Treat. 2023 Feb 7;198(2):335–47. doi: 10.1007/s10549-022-06834-7 (PMC10020257; doi:10.1007/s10549-022-06834-7)
Supplement: Supplementary file 1 — Supplementary file1 (DOCX 21 KB) [file 10549_2022_6834_MOESM1_ESM.docx]

**Validation of a breast cancer risk prediction model based on the key risk factors: family history, mammographic density and polygenic risk**

Richard Allman^1^ (ORCID: 0000-0002-8635-5237)

Yi Mu^2^ (ORCID: 0000-0001-5660-440X)

Gillian S. Dite^1^ (ORCID: 0000-0002-2448-2548)

Erika Spaeth^3^ (ORCID: 0000-0002-5397-3493)

John L. Hopper^4^ (ORCID: 0000-0002-8567-173X)

Bernard A. Rosner^2^ (ORCID: 0000-0001-6907-0056)

1. Genetic Technologies Limited, Fitzroy, Victoria, Australia

2. Channing Division of Network Medicine, Brigham and Women’s Hospital and

Harvard Medical School, Boston, Massachusetts, United States of America

3. Phenogen Sciences Inc, Charlotte, North Carolina, United States of America

4. Centre for Epidemiology and Biostatistics, Melbourne School of Population and Global Health, The University of Melbourne, Parkville, Victoria, Australia

Corresponding author:

Richard Allman

Genetic Technologies Limited

60–66 Hanover St, Fitzroy VIC 3065, Australia

Telephone: +61 3 8412 7005

Email: Richard.allman@gtglabs.com

**Supplementary data**

**Supplementary Table 1.** Performance of 5-year and lifetime risk calculation by the BRISK model using approximated BI-RADS categories for the mammographic density measure.

| **Model** | **OR per SD^a^** | **95% CI** | **P** | **AUC** | **95% CI** |
| --- | --- | --- | --- | --- | --- |
| BRISK 5-year risk | 1.41 | 1.31, 1.52 | <0.001 | 0.631 | 0.611, 0.652 |
| BRISK remaining lifetime risk | 1.49 | 1.39, 1.60 | <0.001 | 0.643 | 0.622, 0.664 |

Note: AUC, area under receiver operating characteristic curve; CI, confidence interval; OR, odds ratio; SD, standard deviation

**Supplementary Table 2.** Summary of classification performance at clinically relevant thresholds for guiding chemoprevention

|  | **Gail** | **BRISK** |
| --- | --- | --- |
| % cases >1.67% | 43.9% | 72.2% |
| % ER+ cases >1.67% | 42.1% | 74.3% |
| % ER− cases >1.67% | 44.3% | 64.3% |
| % controls >1.67% | 42.6% | 53.5% |
|  |  |  |
| % cases > 3% | 8.75% | **51.0 %** |
| % ER+ cases >3% | 8.9% | 53.0% |
| % ER− cases >3% | 9.3% | 39.9% |
| % controls >3% | 7.8% | **32.3%** |
|  |  |  |
| % cases <1% | 10.9% | 14.1% |
| % ER+ cases <1% | 10.3% | 12.7% |
| % ER− cases <1% | 17.9% | 17.1% |
| % controls <1% | 12.2% | **27.8%** |

**Supplementary Table 3.** Summary of classification performance at clinically relevant thresholds for guiding additional screening

|  | **IBIS version 7** | **BRISK** |
| --- | --- | --- |
| % cases >20% | 4.1% | **41.3%** |
| % ER+ cases >20% | 3.9% | 42.9% |
| % ER− cases >20% | 2.8% | 35.8% |
| % stage 1 cases >20% | 4.7% | 42.3% |
| % stage 2+ cases >20% | 2.5% | 38.9% |
| % controls >20% | 2.5% | **22.4%** |
|  |  |  |
| % cases >25% | 0.9% | **33.5%** |
| % ER+ cases >25% | 0.9% | 35.2% |
| % ER− cases > 25% | 0% | 24.5% |
| % stage 1 cases >25% | 1.3% | 34.5% |
| % stage 2+ cases >25% | 0% | 30.5% |
| % controls > 25% | 0% | **16.1%** |
|  |  |  |
| % cases <6% | 10.9% | 17.6% |
| % ER+ cases <6% | 11.9% | 16.9% |
| % ER− cases <6% | 14.2% | 18.9% |
| % stage 1 cases <6% | 11.4% | 18.0% |
| % stage 2+ cases <6% | 12.6% | **14.6%** |
| % controls <6% | 18.1% | 35.3% |

**BRISK model**

**Risk modifier score**

First, calculate a risk modifier score (*risk_mod*) for each woman:

ln(*risk_mod*) = ln(*snprisk*) × 1.03

+ (sqrt(*percent_density*) − 4.14) × 0.12

+ ln(*fh_risk_1*)

+ ln(*fh_risk_2*)

+ −0.04 + ((ln(*bmi*/5) – 1.62) × −0.35) if *menopausal_status*=0

+ 0.05 + ((ln(*bmi*/5) – 1.62) × 0.88) if *menopausal_status*=1

In an alternative version of the risk modifier score, replace the *percent_density* term with:

+ −0.15 if *birads*=0

+ 0.24 if *birads*=1

then:

*risk_mod* = e^ln(^*^risk_mod^*^)^

**Absolute risks**

For the calculation of absolute risks, we used sex- and age-specific (in 5-year groups) population incidences from the Surveillance, Epidemiology and End-Results Program to determine cumulative incidences from birth up to current age (*incid_b*), current age plus 5 years (*incid_b5*), and to age 85 years (*incid_life*). For the calculation of 5-year risks we applied a competing mortality adjustment using sex- and age-specific non-breast cancer mortality to calculate expected survival (*surv*) during the following 5 years.

The absolute 5-year risk was calculated as:

$5yr\_risk=\frac{\left( cumul\_b5 -cumul\_b \right)}{(1-cumul\_b)}$

where

$$cumul\_b=1- e^{-risk\_mod \times incid\_b \times surv}$$

$$cumul\_b5=1- e^{-risk\_mod \times incid\_b5 \times surv}$$

The remaining lifetime risk was calculated as:

$rem\_risk=\frac{\left( cumul\_remlife - cumul\_b \right)}{(1-cumul\_b)}$

where

$$cumul\_b=1- e^{-risk\_mod \times incid\_b}$$

$$cumul\_\_remlife=1- e^{-risk\_mod \times incid\_life}$$

**Polygenic risk score**

The polygenic risk score (PRS) was calculated using published estimates of the odds ratio (OR) per effect allele and effect allele frequency (*p*), assuming independent and additive risks on the logOR scale. For each single-nucleotide polymorphism (SNP), the unscaled population average risk (µ) was calculated as:

$$\mu=\left( 1-p \right)^{2}+2p\left( 1-p \right)OR+p^{2}OR^{2}$$

Next, adjusted risks (which have a population average risk equal to 1) for each SNP were calculated as:

$adjusted\_risk=\frac{OR^{N}}{\mu}$ where N is the number of risk alleles.

The overall PRS (*snprisk*) was then the product of the adjusted risk values for each of the SNPs.

**First-degree family history**

A woman’s first-degree family history risk (*fh_risk_1***)** was based on her age and the number and age of her first-degree relatives with invasive breast cancer. The calculation uses estimates from Table 3, Table 4 and Figure 1 in the Collaborative Group on Hormonal Factors in Breast Cancer paper from 2001.

| **Number of affected first-degree relatives** | **Age (years)** | **Age of affected first-degree relative (years)** | **fh_risk_1** |
| --- | --- | --- | --- |
| 0 | All ages | N/A | 0.94 |
| 1 | <40 | <40 | 4.90 |
| 1 | <40 | 40–49 | 2.90 |
| 1 | <40 | 50–59 | 2.60 |
| 1 | <40 | ≥60 | 1.90 |
| 1 | 40–49 | <40 | 2.94 |
| 1 | 40–49 | 40–49 | 2.34 |
| 1 | 40–49 | 50–59 | 2.14 |
| 1 | 40–49 | ≥60 | 1.64 |
| 1 | 50–59 | <40 | 2.05 |
| 1 | 50–59 | 40–49 | 1.85 |
| 1 | 50–59 | 50–59 | 1.55 |
| 1 | 50–59 | ≥60 | 1.55 |
| 1 | ≥60 | <40 | 1.37 |
| 1 | ≥60 | 40–49 | 1.37 |
| 1 | ≥60 | 50–59 | 1.37 |
| 1 | ≥60 | ≥60 | 1.37 |
| 1 | <35 | Unknown | 2.84 |
| 1 | 35–39 | Unknown | 2.43 |
| 1 | 40–44 | Unknown | 2.00 |
| 1 | 45–49 | Unknown | 1.94 |
| 1 | 50–54 | Unknown | 1.84 |
| 1 | 55–59 | Unknown | 1.74 |
| 1 | 60–64 | Unknown | 1.55 |
| 1 | 65–69 | Unknown | 1.46 |
| 1 | ≥70 | Unknown | 1.37 |
| ≥2 | <50 | At least one <40 | 13.50 |
| ≥2 | <50 | Both ≥40 | 7.80 |
| ≥2 | ≥50 | At least one <40 | 3.90 |
| ≥2 | ≥50 | Both ≥40 | 2.60 |
| ≥2 | <45 | Unknown | 5.00 |
| ≥2 | 45–54 | Unknown | 3.50 |
| ≥2 | 55–64 | Unknown | 2.70 |
| ≥2 | ≥65 | Unknown | 2.30 |

**Second-degree family history**

A woman’ second-degree family history risk (*fh_risk_2***)** was based on her number of second-degree relatives with invasive breast cancer.

| **Number of affected second-degree relatives** | **Age (years)** | **fh_risk_2** |
| --- | --- | --- |
| 0 | All ages | 0.97 |
| 1 | <40 | 1.7 |
| 1 | 40–49 | 1.4 |
| 1 | 50–59 | 1.3 |
| 1 | ≥60 | 1.2 |
| ≥2 | <55 | 2.1 |
| ≥2 | ≥55 | 1.6 |

**Mammographic density**

The *percent_density* term is a continuous measure of the percent dense area as measured using computerised or computer-assisted methods. The alternate *birads* term is 0 if a radiologist has classified the mammogram as A or B, and 1 if a radiologist has classified the mammogram as C or D.

**Body mass index**

Body mass index (*bmi*) is a continuous measure calculated as weight (kg) divided by the square of height (m^2^).

**Menopausal status**

Menopausal status (*menopausal_status*) is coded as 0 if a woman is pre-menopausal or peri-menopausal, and 1 if a woman is post-menopausal.

Collaborative Group on Hormonal Factors in Breast Cancer. Familial breast cancer: collaborative reanalysis of individual data from 52 epidemiological studies including 58,209 women with breast cancer and 101,986 women without the disease. *Lancet.* 2001; 358(9291): 1389–1399. doi: 10.1016/S0140-6736(01)06524-2

Hopper JL, Dite GS, MacInnis RJ, et al. Age-specific breast cancer risk by BMI and familial risk: prospective family study cohort (ProF-SC). *Cancer Res*. 2018; 20(1): 132. doi: 10.1186/s13058-018-1056-1
